# Mental and Body Health: The Association between Psychological Factors, Overweight, and Blood Pressure in Young Adults

**DOI:** 10.3390/jcm11071999

**Published:** 2022-04-02

**Authors:** Giuseppe Forte, Francesca Favieri, Mariella Pazzaglia, Maria Casagrande

**Affiliations:** 1 Department of Psychology, Sapienza University of Rome, Via dei Marsi 78, 00185 Roma, Italy; francesca.favieri@uniroma1.it (F.F.); mariella.pazzaglia@uniroma1.it (M.P.); 2 Body and Action Laboratory, IRCCS Santa Lucia Foundation, Via Ardeatina 306, 00179 Rome, Italy; 3 Department of Dynamic and Clinical Psychology and Health Studies, Sapienza University of Rome, 00185 Roma, Italy; maria.casagrande@uniroma1.it

**Keywords:** cardiometabolic risk factors, blood pressure, weight condition, depression, anxiety, emotional dysregulation

## Abstract

Comorbidity between cardiometabolic risk factors and major mental health disorders is a public health concern. The close interconnection between the mental and physical aspects of health precludes considering each condition separately. Accordingly, this study sought to explore the interrelationships between psychological factors, overweight, and blood pressure in young adults. One hundred and forty-five young adults participated in the study and were classified according to two independent characteristics: weight condition (normal weight, overweight) and blood pressure (low blood pressure, high blood pressure). Anxiety, depression, and emotional dysregulation were assessed. The results confirmed certain associations, highlighting how cardiometabolic risk factors, such as blood pressure and body mass index, were associated in different ways with mental health, although an interaction between the variables was not reported. In particular, a relationship between body mass index and depression and between anxiety and blood pressure was detected.

## 1. Introduction

Comorbidity between cardiometabolic risk factors and major mental health disorders is a public health concern. High rates of cardiometabolic risk factors, particularly excessive body weight and high blood pressure (BP), contribute greatly to an increased incidence of cardiovascular diseases among individuals with mental health problems, reducing their life expectancy by 10 to 20 years [1,2,3]. Cardiometabolic risk factors and overt cardiovascular diseases in individuals with mental health disorders are closely linked to the individuals’ lifestyles (e.g., diet, physical activity, and smoking habits). Further, several neurobiological hypotheses concerning potential shared mechanisms have been proposed to explain the interactions between mental and cardiometabolic disease [4]. For example, an alteration in the systems involved in homeostatic adjustments (i.e., the hypothalamic–pituitary–adrenal [HPA] axis) has been hypothesized to be involved in major depressive disorder, and alterations in the physiological homeostasis of neuroendocrine, metabolic, and microbiome systems have been suggested to be mediators of the interaction between mental and cardiometabolic health [4]. However, the interrelation among these factors is complex, making it difficult to clarify the causal relationships between them and suggesting a bidirectional association between psychological status, cardiometabolic activation, and physical health. 

Considering BP alterations, which are one of the main risk factors for cardiovascular pathologies, some studies have focused on the association between hypertension and psychological distress (e.g., anger, anxiety, emotional dysregulation, and depressive symptoms), producing mixed findings [5,6,7,8,9]. Generally, studies have confirmed an association between psychological variables (e.g., coping strategies and alexithymia) and hypertensive diagnoses at different grades of severity in adult populations [5,8]; this may be explained by distress-related physiological alterations having a direct (i.e., psychophysiological changes) or indirect (i.e., consequences of labeling) effect on BP. Although psychosomatic research on BP has mainly focused on hypertension, some studies have indicated that low BP is also associated with somatic and psychological symptoms [10,11,12,13,14], confirming body–mind interaction regarding both hyper- and hypotension. 

Being overweight, which is an additional risk factor for cardiometabolic health status, appears to be associated with elevated levels of mental distress [15,16], and there is a positive correlation between weight increase and severity of mental issues [17]. However, the findings are inconsistent. Some studies have found no relationship [18] or an inverse relationship [19]. Longitudinal studies have suggested that obesity may be both a cause and a consequence of mental distress, creating a vicious circle [15,16]. On the one hand, obesity-related stigmatization may result in high mental distress [20,21,22]; on the other hand, inappropriate eating behaviors (e.g., emotional eating) may lead to obesity in individuals with poor mental health and maladaptive coping strategies [23]. Moreover, the effect of antipsychotic medication for the treatment of psychiatric disorders (e.g., schizophrenia [24]) is a risk factor for severe weight gain. If associated with severe adiposity, both conditions can generate cardiovascular damage. In general, the inconsistent study results suggest that the relationship between being overweight and having a mental health condition is complex and remains unclear.

The close interconnection between the mental and physical aspects of health precludes considering each condition separately. However, in an attempt to define the nature of the association between cardiometabolic risk factors and mental disorders, it could be useful to focus on non-pathological conditions of mental distress (e.g., depressive symptomatology, anxiety, and emotional dysregulation) in the general healthy population and to identify the relations between these dimensions earlier in a person’s life, especially before the onset of a medical or psychological diagnosis. Young adults are an excellent and convenient sample for analyzing this relationship because young adulthood is a period of life during which early symptomology can be seen for conditions such as unhealthy weight gain, high BP, and some mental problems [25]. Young adults are exposed to both eustress and distress, which differentially impact the brain system (especially the limbic system, which influences physical homeostasis and regulates autonomic nervous system response, emotions, behavior, and caloric intake) [26,27]. Accordingly, this study aims to explore the interrelationships between certain psychological factors, being overweight, and BP in young adults.

## 2. Materials and Methods

### 2.1. Participants

One-hundred and forty-five healthy young adults from 20 to 30 years old (89 females, 56 males; mean age = 23.50 (2.85); mean years of education = 16.33 (1.96)), recruited voluntarily through public notices, participated in the study. The participants were classified considering two independent characteristics: weight condition (normal-weight, overweight; according to WHO classification via body mass index—BMI) and blood pressure (low blood pressure, high blood pressure; according to the European guidelines for the classification of blood pressure [28]). A history of psychiatric or psychological diagnosis and/or chronic medical conditions (e.g., diabetes, metabolic syndrome, hypertension) were adopted as exclusion criteria.

### 2.2. Measures

Physiological Measures

Systolic (SBP), diastolic blood pressure (DBP) and heart rate were recorded using an automatic electronic sphygmomanometer validated for self-measurement (“Personal Check” PIC) according to European guidelines criteria [26]. Blood pressure has been categorized as low (SBP lower than 120 mm Hg and/or DBP lower than 80 mm Hg) or high (SBP higher than or equal to 120 Hg and/or DBP higher than or equal to 80 mm Hg [28]). However, if the SBP is less than 100 mm Hg and/or the DBP is less than 60 mm Hg, we have hypotension. The preclinical forms of hypertension and hypotension (blood pressure tends to be high or low) in young adults represent risk factors for developing overt conditions of hypertension and hypotension. After a 5 min rest in which participants were seated on a comfortable chair with their backs resting on their backrests, they were required to keep silent, and then the three recommended measurements were carried out on the non-dominant arm, at about 2 min from each other.

A balance and a meter were used to measure the weight and height of the participants. Weight and height were used to calculate the body mass index (BMI), an indirect estimate of body fatness. BMI was obtained by dividing weight (in kilograms) by height (in meters squared). Individuals with a BMI lower than or equal to 25 kg/m^2^ were included in the normal-weight group; participants with a BMI higher than or equal to 25 kg/m^2^ were included in the overweight group, including both overweight and obesity conditions according to the WHO classification [29]. 

### 2.3. Socio-Demographic and Anamnestic Information

Demographic data (age, gender, marital status, years of education), lifestyles (smoking, alcohol consumption), and medical and psychiatric information were collected for each patient by face-to-face interview. 

### 2.4. 20-Item Toronto Alexithymia Scale (TAS-20)

TAS-20 [30] is a self-report questionnaire that evaluates alexithymia. It includes 20 items on a 5-point Likert scale (1 = strongly disagree, 5 = strongly agree). The test also enables assessment of three different facets of alexithymia: difficulty identifying feelings (DIF), difficulty describing feelings (DDF), and externally oriented thinking (EOT). The scores range from 20 to 100 and provide both categorical and continuous information. In categorical classification, three different levels of alexithymia are considered: non-alexithymic (scores below 51); moderately alexithymic (scores between 51 and 60); alexithymic (scores above 60).

### 2.5. State-Trait Anxiety Inventory (STAI)

The trait scale of the State-Trait Anxiety Inventory (STAI) [31] is a self-assessment questionnaire to evaluate state and trait anxiety on a 4-point Likert scale (1 = not at all; 4 = very much). Higher scores on the STAI indicate greater anxiety levels. According to the aim of the study, only trait anxiety (20 items) was assessed.

### 2.6. Beck Depression Inventory (BDI)

The Beck Depression Inventory (BDI) [32] was used to assess depressive symptoms. It contains 21 multiple-choice items investigating different depressive symptoms (e.g., hopelessness, irritability), cognitions (e.g., guilt, feelings of being punished), and physical symptoms (e.g., fatigue, weight loss) related to depression. 

### 2.7. Procedure

The research was conducted according to the Declaration of Helsinki and was approved by the Ethics Committee of the Department of Dynamic and Clinical Psychology and Health Studies of the Sapienza University of Rome (protocol n. 0001166). An experimenter trained in the research procedure administered the questionnaire and collected the physiological data in a laboratory setting. After signing the informed consent form, the participants were subjected to blood pressure recordings, and then weight and height were measured. Then, the participants were submitted for a socio-demographic and anamnestic interview, and completed all the questionnaires. The whole procedure, lasting 40 min, took place in a quiet environment with a comfortable temperature.

### 2.8. Data Analysis

Descriptive data analyses were performed, considering the sex (males and females) and the weight condition (normal weight, overweight) of participants. Univariate analyses of variance (ANOVAs) were carried out to evaluate participant differences in age, years of education, and physiological measurements. The Chi-square test was adopted for nominal variables. Mixed ANOVAs were carried out to assess the differences between the groups, considering sex and body weight condition as independent variables and psychological assessments (BDI, STAI, TAS-20) as dependent variables. 

## 3. Results 

### 3.1. Demographic Characteristics 

All descriptive data relating to blood pressure and weight condition classification are reported in Table 1 and Table 2. 

### 3.2. Psychological Aspects 

#### 3.2.1. Alexithymia

The ANOVAs on the subscales of TAS-20, which considered blood pressure and weight condition as independent between-factors variables showed that participants with high BP reported significantly higher scores than participants with low BP in the externally oriented thinking subscale (F_1137_ = 12.7; *p* = 0.001; pn^2^ = 0.12). However, no other significant effect or interaction was observed for TAS-20 subscales or the TAS-20 global score (see Table 3 and Table 4). 

#### 3.2.2. Depression

The ANOVA on the BDI score as an index of depressive symptoms indicated a significant effect of weight condition (F_1137_ = 15.78; *p* = 0.0001; pn^2^ = 0.15). Participants with overweight reported higher depression states than individuals with normal weight (see Table 3). No differences were reported considering blood pressure (F_1137_: 2.78; *p* = 0.10) or blood pressure x weight condition interaction (F_1137_: 1.40; *p* = 0.23). 

#### 3.2.3. Anxiety

The ANOVA on the STAI score as an index of trait anxiety symptoms indicated significant differences considering weight condition (F_1137_ = 3.83; *p* = 0.04; pn^2^ = 0.05), and a significant effect of blood pressure (F_1137_ = 14.30; *p* = 0.0001; pn^2^ = 0.13). Participants with low blood pressure reported higher trait anxiety than individuals with high blood pressure (see Table 4). No significant difference was reported considering the blood pressure x weight condition interaction (F < 1; *p* = 0.37). 

## 4. Discussion

The statement “Mens sana in corpore sano” captures the idea that bodily health is essential to foster and maintain mental and psychological well-being and vice versa. The mind and body must be considered inseparable elements, representing two distinct aspects of human beings that influence each other. Accordingly, the main aim of this study was to determine the interconnection between both physical condition (i.e., being overweight and high BP) and mental (i.e., emotional dysregulation, depressive symptomatology, and anxiety) risk factors for health status, focusing on early stages of their association (i.e., their preclinical occurrence in the young adult population). Anxiety levels, alexithymia (as a pattern of emotional dysregulation) and depressive symptomatology were considered indices of emotional distress. One of the first results of this study concerns the role of alexithymia as a pattern of emotional dysregulation. Although there was a general association between emotional dysregulation and being overweight [33,34] or experiencing hypertension [9,35], and although the average trends of this study suggested similar results, this association was generally not confirmed in our sample, except for one facet of TAS-20. Higher externally oriented thinking was observed in individuals with high BP, suggesting an alteration in some emotional regulation aspects associated with poor mentalization abilities. Our previous study [8] found that the severity of hypertension modulated the association between alexithymia and hypertension. Patients diagnosed with uncontrolled hypertension (patients who cannot maintain control of their BP despite following pharmacological treatment) reported higher levels of alexithymia, characterized by difficulty in identifying and describing their feelings. Alexithymia, a personality trait characterized by difficulties in identifying and describing feelings, has been associated historically with psychosomatic disorders [36,37]. However, the mechanisms underlying the link between emotional dysregulation and psychosomatic disorders are unclear. Identifying an early association between BP alterations and alexithymic patterns could provide interesting insights into the analysis of this relationship. 

Considering the results regarding depressive symptoms, despite a meta-analysis of prospective studies that found that depressive symptoms predicted a 42% increased risk of hypertension [38], our results did not confirm an association with high BP in young adults. However, an association was found between depressive symptomatology and overweight condition. The mechanisms linking BMI and depression are likely to be complex, as several studies have indicated that each may induce or worsen the other [39,40]. A high BMI could influence the risk of depression via biological mechanisms (e.g., inflammation or dysregulation of hormonal systems) or have secondary negative psychological effects (e.g., on self-image), which could generate negative affectivity [40]. Recent evidence suggests an extensive genetic overlap between BMI and affective disorders, indicating a complex interplay of metabolism-related gene pathways in the pathophysiology of major depression [41]. Depression could also influence the risk of high BMI via unhealthy behavioral lifestyles generated as an attempt to cope with negative affect and depressive symptoms (e.g., physical inactivity, unhealthy diet, and overeating) [39]. According to the current nosographic classification, depression is associated with both increased and decreased food intake and increased or decreased physical activity. Therefore, it seems logical that increased levels of depression are associated with weight gain [42], indicating a possible U-shaped association that should be further investigated. Our results suggest an association between depression and BMI, independent of BP, suggesting that this interaction in young adults should be controlled as a preventative measure before it becomes an overt disorder. Future studies should consider larger samples and longitudinal assessments to better understand and explain this relationship. Finally, regarding anxiety, heterogeneous results on its relationship with both weight status [43,44] and BP [12,45] have been reported in previous studies. Regarding BP, a meta-analysis by Pan et al. [4] showed that studies had found anxiety symptoms to be an independent risk factor for hypertension. Generally, anxiety increases systemic vascular resistance, sympathetic activity, and blood lipids. The short-term (i.e., white coat effect) [46,47] and long-term (i.e., nocturnal hypertension) [48] effects of anxiety previously reported in the literature may influence the occurrence of hypertension, decrease vascular variability and stimulate sympathetic nervous outflow and the vasovagal reflex [49,50], thus establishing vascular resistance that leads to hypertension [51]. In contrast, our results suggest a relationship between low BP and anxiety in healthy young adults. This finding might seem counterintuitive; however, previous studies have shown similar results, including cohort studies of large sample size. These studies have demonstrated a relationship between low BP and poor mental health and indicated that anxiety and depression could be associated with a decrease in BP, particularly when a high symptom level is observed across multiple decades [12,52,53]. Therefore, the following question arises: What mechanisms are involved in the relationship between low BP and anxiety? A physiological explanation could be the overexpression of neuropeptide Y, which seems to reduce BP and was observed in both individuals with anxiety and with depression [54,55,56,57]. Two main neuroendocrine systems seem to play a pivotal part in the interaction between BP and anxiety: the hypothalamic–pituitary–adrenocortical axis and the renin–angiotensin system. The association between hypotension and mental health contrasts with the traditional view that hypertension may be etiologically linked to personality or certain mental state abnormalities. However, recent studies on low BP have mainly sought to identify correlations between BP and psychological and neurological conditions. Although the results are mixed, many studies have suggested that both higher and low BP are associated with cognitive impairment (for a review [57,58]) and mental and neurological health problems [59,60,61,62,63], suggesting low cerebral perfusion as a possible biological mechanism for cognitive impairment. Another explanation is derived from a behavioral perspective: after periods of intense anxiety, which is characterized by physiological hyperactivation, the body is left feeling very fatigued, and this can contribute to low BP. Accordingly, this study mainly focused on analyzing trait anxiety rather than the state of anxiety. However, the dominant attitude regarding low BP in the clinical field is that chronic low BP does not produce symptoms and that the prognosis is better than in individuals with higher BP. For these reasons, the study of low BP is more controversial and more research is needed on this topic. Although a robust body of research has highlighted the association between psychopathological conditions and cardiometabolic risk factors (e.g., being overweight and high BP), this is the first study to analyze mental health in young adults with preclinical forms of mental health distress. 

Nevertheless, the present study results confirm some previous evidence [5,6,9,10,18]. Further studies are necessary due to the limitations of this investigation. First, the cross-sectional nature of the study did not allow defining a causal relationship and identifying the trend of the highlighted relationship in lifespan. Second, the small sample size limits the generalizability of the results, specifically considering certain possible confounding variables (e.g., gender and lifestyle). Another limitation that can be considered in further studies is the absence of clinical groups diagnosed with mental disorders or severe cardiometabolic alterations. Analyzing these populations could allow identifying differences and similarities and defining psychological markers of cardiac health status. Moreover, other lifestyle factors (e.g., physical exercise, diet habits) should be further analyzed in their relationship with these physiological alterations and their impact on psychological well-being.

## 5. Conclusions

From the mind and body integration perspective, this study investigated the interrelationship among BP, BMI, and psychological aspects of health (e.g., depression, anxiety, and emotional dysregulation) in a sample of healthy young adults. The findings partially confirm this association, highlighting how cardiometabolic risk factors (e.g., BP and BMI) associate differently with mental health. In particular, a relationship between BMI and depression was observed, while BP was associated with anxiety. Our findings have potential consequences for clinical studies on both non-pharmacological and pharmacological treatments of BP, which should take common mental symptoms of participants into account. Although the results are interesting, further studies with larger samples and longitudinal investigations are needed.

## Figures and Tables

**Table 1:** Characteristics of normal-weight and overweight groups.

	Normal Weight	Overweight	X^2^/F	*p*
N (M/F)	107 (37/70)	38 (20/18)		
Age	23.07 (2.78)	24.73 (2.68)	10.21	0.002
Years of Education	16.26 (1.98)	16.62 (1.84)	<1	0.33
BMI (kg/m^2^)	21.08 (1.95)	27.18 (2.85)	208.49	0.0001
SBP	115.95 (11.08)	119.86 (10.76)	3.38	0.07
DBP	71.05 (8.03)	74.25 (7.28)	4.43	0.04
HR	77.26 (12.91)	75.45 (11.03)	<1	0.46
Lifestyle habits, *n* (%)			X^2^	*p*
Smoking Habits *			<1	0.69
Yes	40 (37.7)	15 (41.7)		
No	66 (62.3)	21 (58.3)		
Caffeine Consumption *			<1	0.82
Yes	82 (77.4)	27 (75.0)		
No	24 (22.6)	9 (25.0)		
Alcohol Consumption *			<1	0.83
Yes	61 (57.5)	20 (55.6)		
No	45 (42.4)	16 (44.4)		

M: males; F: females; SBP: Systolic Blood Pressure; DBP: Diastolic Blood Pressure; HR: Heart Rate. * three missing data.

**Table 2:** Characteristics of the groups with high and low blood pressure.

	Low Blood Pressure	High Blood Pressure	X^2^/F	*p*
N (M/F) *	76 (13/63)	65 (41/24)		0.0001
Age	22.81 (2.22)	23.98 (3.02)	6.94	0.009
Years of Education	16.35 (1.63)	16.21 (2.15)	<1	0.66
BMI (kg/m^2^)	21.86 (2.72)	23.58 (3.93)	9.25	0.003
SBP	108.94 (7.34)	126.31 (6.43)	219.26	0.0001
DBP	67.85 (6.11)	76.56 (7.26)	59.80	0.0001
HR	75.09 (11.68)	78.91 (13.11)	3.21	0.07
Lifestyle habits, *n* (%)			<1	0.59
Smoking Habits				
Yes	31 (41.4)	24 (36.9)		
No	44 (58.6)	41 (63.1)		
Caffeine Consumption			<1	0.89
Yes	57(76.0)	50 (76.9)		
No	18 (24.0)	15 (23.1)		
Alcohol Consumption			2.18	0.14
Yes	38(51.0)	41 (63.1)		
No	37 (49.0)	24 (36.9)		

M: males; F: females; BP: Systolic Blood Pressure; DBP: Diastolic Blood Pressure; HR: Heart Rate, * Four missing data.

**Table 3:** Mean and standard deviation of the psychological dimensions in normal weight and overweight groups.

	Normal Weight	Overweight	F	*p*
Alexithymia (TAS-20)				
Difficulty in identifying feelings	13.13 (5.00)	13.88 (6.01)	1.13	0.29
Difficulty in describing feelings	12.14 (5.31)	13.71 (5.76)	1.28	0.25
Externally oriented thinking	14.92 (3.61)	15.51 (4.98)	<1	0.87
Total score	40.21 (11.38)	43.10 (11.95)	<1	0.33
Depression (BDI)	7.66 (6.26)	13.35 (8.44)	15.78	0.0001
Anxiety (STAI)	45.34 (4.44)	46.64 (6.13)	3.83	0.06

TAS-20: Toronto Alexithymia Scale- 20 items; BDI: Back Depression Inventory; STAI: Stait-Trait Anxiety Inventory.

**Table 4:** Mean and standard deviation of the psychological dimensions in low blood pressure and high blood pressure groups.

	Low Blood Pressure	High Blood Pressure	F	*p*
Alexithymia (TAS-20)				
Difficulty in identifying feelings	13.78 (5.17)	12.95 (5.93)	2.36	0.12
Difficulty in describing feelings	11.95 (5.51)	13.20 (5.41)	<1	0.47
Externally oriented thinking	13.83 (3.38)	16.26 (4.26)	12.16	0.0001
Total score	39.57 (11.05)	42.41 (11.96)	<1	42
Depression (BDI)	9.82 (7.85)	8.86 (6.99)	2.78	0.09
Anxiety (STAI)	47.47 (4.63)	44.10 (4.80)	14.30	0.0001

TAS-20: Toronto Alexithymia Scale- 20 items; BDI: Back Depression Inventory; STAI: Stait-Trait Anxiety Inventory.
